# Annotations

**Published:** 1893-06-24

**Authors:** 


### ANNOTATIONS.

**Mr. Alabone and his Editorial Friends.**

There is some reason to hope that the quarrel as fought out before Mr. Justice Wright, between Mr. Edwin Alabone, formerly a registered medical practitioner, and his quondam clerk, Henry E. M. Morton, will bring a good many facts to light which may open the eyes of the public. Alabone has been a most foolish man. He began life well; is a person of liberal culture; and if he had been willing to endure with patience the tedium of waiting for success, which is practically every medical man's experience, there is no doubt he might now have been a prosperous and a happy man. Instead of that he took to the practices of the puffing advertiser. He seems to have persuaded large numbers of the public that he had a "specific" for consumption. What his "specific" actually was we now see, and its worthlessness is patent to everybody. For years Alabone had a remarkable run of prosperity. Whilst all the other doctors in his neighbourhood were toiling as only doctors can toil, for little more than a bare living, Alabone was dashing up and down in a smart carriage drawn by a pair of high-stepping bays, the equal of the smartest in the Row. Of course the local doctors declined to have anything to do with him; and equally, of course, many of the wealthiest people in the neighbourhood admired Alabone and sneeringly declared that the other doctors were animated by the meanest jealousy because, as they said, he could cure consumption and they could not. The story is as old as the hills. It will be repeated again a hundred times over before the present century is out. In the particular case of Alabone the self-puffer has been brought to book, and no doubt his brief day of prosperity is over. We must now hope that the Public Prosecutor will go thorougly into the case, and that the public for once may see the sordid facts as they are. Alabone and his clerk belong to the same class, and their exposure of one another may be of service in unearthing a few more of their peculiar fraternity. Certain newspapers come rather meanly out of the business. Some "religious" newspapers have most unscrupulous consciences. The editors apparently have little faith in future punishment, however ardent may be their hopes of future reward. But they show a touching confidence in the virtue of "a bird in the hand."

**The Brighton Life Table.**

The Brighton Life Table, just completed by Dr. Arthur Newsholme, medical officer of health for the borough, brings out a good many interesting things. Among the rest it shows us that women live much longer than men at Brighton—as, of course, they do elsewhere. It does not tell us whether this is due to the fact that the average Brighton woman has a nice easy time of it dawdling about the seashore, whilst her husband, her brothers, and her sons have to rush up and down between London and their home at the sea at the rate of fifty miles an hour morning and evening. A fact of very peculiar interest to the gentler sex is that the average woman lives from seven to ten years longer at Brighton than she does at Manchester, and from four to five years longer than she does in England and Wales generally. This latter statement we should have been inclined to question if we had had less confidence in Dr. Newsholme as a statician and an impartial scientist. There is less difference between the relative longevity of men at Brighton, at Manchester, and throughout England and Wales. Readers must not hastily conclude that because Brighton is healthier than Manchester, and because its longevity is in excess of that of the whole of England and Wales, therefore it is the healthiest town in all England and Wales. That would be very inadequate reasoning. Dr. Newsholme, in selecting Manchester for comparison, has selected one of the unhealthiest towns in the kingdom, and in selecting England and Wales has selected all the large as well as the small towns, including London, Birmingham, Liverpool, Leeds, and all the manufacturing centres. It would have been more satisfactory if, in addition to the places actually selected, a comparison had also been instituted between such towns as Bournemouth and Brighton, or Tunbridge Wells and Brighton. No doubt Brighton ranks high among health resorts. But it is probable that there are other towns in the British Isles which rank quite as high, or even higher. The special pleader, however distinguished and scientific, always requires to be watched.

**A Terrible Warning to Nurses.**

The *Times* of Saturday last reported the facts of an inquest held concerning the death of Frederick Vincent Malim, aged 12 years, the son of Mr. Aubrey Vincent Malim, solicitor and Town Clerk of Grantham, who died at Bengeo College, Hertford, from a dose of carbolic acid, administered to him by a certificated nurse instead of medicine. The nurse who carelessly made this fatal mistake was Ada Barnard, and she had been sent out to Bengeo College from St. John the Divine Nursing Institute, South Kensington, to take charge of several boys who were warded, suffering from measles. Nurse Barnard, on being examined, stated that she was properly certificated. . . . She received the bottle of carbolic acid from the matron at about eight o'clock in the evening. The acid was for use in the steam kettle. She entered the dormitory with the intention of placing the bottle out of the way. One of the boys called for a drink, and she put the bottle down on the table while she attended to him. She forgot afterwards to remove it. At ten o'clock she went to bed, and was roused about one by the boy's coughing. She sprang out of bed and took up a bottle from the table to give him some mixture. She poured something into a glass, and, without looking at it, gave it to the deceased. Directly after he said it felt like liquid fire. It then flashed across her mind that she had made a mistake, and she examined the vial, which confirmed her fears. She gave the boy some milk, and ran for Nurse McKay. She was not really awake until after she had given deceased the acid. We may complete this confession of facts by adding the verdict of the jury. "We find," said the spokesman of the jury, "that the deceased died from administration of carbolic acid in mistake for cough mixture, by Nurse Barnard, under circumstances which satisfy the jury that there was gross negligence and carelessness on her part. But the jury think she was not sufficiently awake at the time to make her criminally responsible." A case like this is too distressing, and, to the nurse concerned, too disgraceful for comment. The jury apparently could see no extenuating circumstance, nor indeed can we. There are some nurses, happily but a few, who care no more for the sufferings of their patients, no more for the issues of life and death than they care for the blowing of the wind. We have seen such nurses at work. Their indifference, their carelessness, their stupid selfishness are shocking and intolerable. What certificate can take the place of conscience and intelligence? We would willingly have spared ourselves the pain of recording this case, and conscientious nurses the shame of reading the record. But we are anxious that every nurse in the kingdom may have these most painful facts graved deeply on her mind in order that there may never again be a home bereaved of child or grown-up person by the "gross negligence and carelessness" of a nurse.

